# Nkx2.7 and Nkx2.5 Function Redundantly and Are Required for Cardiac Morphogenesis of Zebrafish Embryos

**DOI:** 10.1371/journal.pone.0004249

**Published:** 2009-01-22

**Authors:** Chi-Tang Tu, Tzu-Ching Yang, Huai-Jen Tsai

**Affiliations:** Institute of Molecular and Cellular Biology, National Taiwan University, Taipei, Taiwan; Katholieke Universiteit Leuven, Belgium

## Abstract

**Background:**

*Nkx2.7* is the *tinman*-related gene, as well as orthologs of *Nkx2.5* and *Nkx-2.3*. Nkx2.7 and Nkx2.5 express in zebrafish heart fields of lateral plate mesoderm. The temporal and spatial expression patterns of *Nkx2.7* are similar to those of *Nkx2.5*, but their functions during cardiogenesis remain unclear.

**Methodology/Principal Findings:**

Here, Nkx2.7 is demonstrated to compensate for Nkx2.5 loss of function and play a predominant role in the lateral development of the heart, including normal cardiac looping and chamber formation. Knocking down Nkx2.5 showed that heart development was normal from 24 to 72 hpf. However, when knocking down either Nkx2.7 or Nkx2.5 together with Nkx2.7, it appeared that the heart failed to undergo looping and showed defective chambers, although embryos developed normally before the early heart tube stage. Decreased ventricular myocardium proliferation and defective myocardial differentiation appeared to result from late-stage up-regulation of *bmp4*, *versican*, *tbx5* and *tbx20*, which were all expressed normally in hearts at an early stage. We also found that *tbx5* and *tbx20* were modulated by *Nkx2.7* through the heart maturation stage because an inducible overexpression of *Nkx2.7* in the heart caused down-regulation of *tbx5* and *tbx20*. Although heart defects were induced by overexpression of an injection of 150-pg *Nkx2.5* or 5-pg *Nkx2.7* mRNA, either *Nkx2.5* or *Nkx2.7* mRNA rescued the defects induced by Nkx2.7-morpholino(MO) and Nkx2.5-MO with Nkx2.7-MO.

**Conclusions and Significance:**

Therefore, we conclude that redundant activities of Nkx2.5 and Nkx2.7 are required for cardiac morphogenesis, but that Nkx2.7 plays a more critical function, specifically indicated by the gain-of-function and loss-of- function experiments where *Nkx2.7* is observed to regulate the expressions of *tbx5* and *tbx20* through the maturation stage.

## Introduction

The heart is the first organ to form and function in the vertebrate embryos. The functional heart is formed after 72 hours post-fertilization (hpf). During heart developmental processes, cell fate determination, specification, differentiation and migration are all involved. In view of this complexity, investigators have used model systems, including *Drosophila*, *Xenopus*, zebrafish, chicken, and mouse, in an attempt to understand the molecular regulatory network of cardiogenesis [1]–[4]. These efforts have produced two key findings important to the hypothesis of this paper: 1) that the members of the homeobox gene family are important for heart development [5] and 2) that the NK-class of homeodomain proteins plays key roles in the establishment of myogenic lineages [6]–[11].

Among the homeobox genes, the vertebrate homologs of *tinman* have been cloned in many species and are highly conserved in the homeodomain structures and expression patterns. For example, *Drosophila tinman* is involved in the specification of the heart primordial cells. More specifically, in *tinman* null mutants, the heart-like dorsal vessel is absent, indicating that *tinman* is a critical factor in *Drosophila* heart-like formation [6]. The mouse *tinman* homolog is *Nkx2.5*, and *Nkx2.5*-knockout mice exhibit the formation of a primitive heart tube, which expresses most cardiac markers with the exception of *mlc2v*. The *mlc2v* marker, however, displays a thinning ventricular myocardium and, therefore, cannot undergo heart looping [7]. Unlike *Drosophila tinman*, mouse *Nkx2.5* is required for murine normal heart morphogenesis, but it is not essential for the cell fate determination of heart precursors. This suggests that there may be an *Nkx* homolog that compensates for the loss of function of *Nkx2.5* in mouse embryos. The most likely homolog of mouse *Nkx2.5* is mouse *Nkx2.6* which shares identical expression patterns at pharyngeal endoderm and at heart with *Nkx2.5* [12]. Targeted disruption of *Nkx2.6* does not cause any abnormality, either in the pharynx or in the heart [13]. In *Nkx2.5^−/−^Nkx2.6^−/−^* double knockout mice, the development of pharynx is totally abolished. Therefore, Tanaka *et al*. (2001) demonstrated that *Nkx2.5* can compensate for the function of *Nkx2.6* in the pharyngeal endoderm [14].

Many *tinman* (*Nkx2.3*, *2.5*, *2.6*, *2.7*, *2.8*) homologs of chicken, *Xenopus* and zebrafish have been isolated [10], [15]–[21]. The example cited above, mouse Nkx2.5/ Nkx2.6, suggests a redundant activity for loss of a single Nkx homolog function. In chick, *cNkx2.8* expression partially overlaps with *cNkx2.5* and *cNkx2.3*. This is seen in the onset of *cNkx2.8*, which appears at heart primordia and retains its expression until primary heart tube formation. After heart tube looping, however, *cNkx2.8* is no longer expressed, and *cNkx2.3* initiates expression at this stage and continues to express until adulthood [22]. Overexpression of cNkx2.8 can transactivate a minimal promoter containing the *cis*-acting element for Nkx2.5 binding. In addition, cNkx-2.8 and serum response factor can co-activate a minimal cardiac α-actin promoter like cNkx2.5 [21]. Still, there is no direct evidence to demonstrate that cNkx2.8 can compensate for cNkx2.5 loss of function in early development of chick heart.

In *Xenopus*, both *XNkx2.3* and *XNkx2.5* are expressed in cardiac mesoderm and pharyngeal endoderm [23]. Injection of *XNkx2.3* or *XNkx2.5* mRNA causes an abnormally enlarged heart [9]. Although the *XNkx2.5* and *XNkx2.3* dominant-negative engrailed-fusion repressor shows a reduced number of heart cells, co-injection of both EnHD constructs appears to result in an even more serious decrease of heart cells. This suggests that *XNkx2.5* and *XNkx2.3* act redundantly during heart formation [24].

In zebrafish, *Nkx2.7* is the *tinman*-related gene, as well as orthologs of *Nkx2.5* and *Nkx2.3*. The transcription of zebrafish *Nkx2.7* is earlier than that of *Nkx2.5* in cardiac mesoderm, and *Nkx2.7* is also expressed at pharyngeal endodermal precursors. The temporal and spatial expression patterns of *Nkx2.7* are similar to those of *Nkx2.5* [19]. While knockdown of *Nkx2.5* in zebrafish embryos displays no obvious defects in heart, overexpression of *Nkx2.5* did show an enlarged heart and even caused dorsoventral axial defects [10]. Since it is not known which stages of cardiac development or which pathways are affected by the Nkx family of homologs, it may be necessary to knock down more than one of these genes to discover their dual functions. Meanwhile, the precise role played by *tinman* homolog genes in vertebrate cardiac development remains to be clarified, particularly the dual roles played by *Nkx2.5* and *Nkx2.7* in the cardiogenesis of zebrafish embryos. In this study, we clearly demonstrate that, while *Nkx2.5* and *Nkx2.7* play redundant roles in the differentiation of cardiomyocytes, *Nkx2.7* has a more critical function, specifically indicated by the gain-of-function and loss-of-function experiments where *Nkx2.7* is observed to regulate the expressions of *tbx5* and *tbx20* through the heart tube stage.

## Results

### *Nkx2.5* and *Nkx2.7* double knockdown results in serious zebrafish embryo heart defects

**Figure 1:**
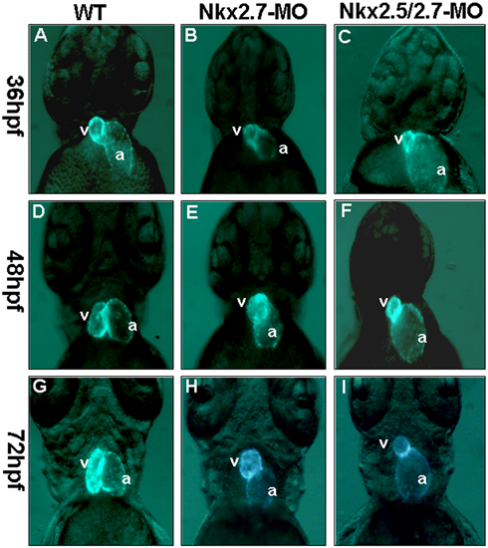
The defective phenotypes of zebrafish embryo heart injected with Nkx2.5-MO, Nkx2.7-MO and Nkx2.5/2.7-MO. Eight nanograms of MO were injected into one-cell stage embryos derived from transgenic line Tg (*cmlc2*::GFP) to knock down the Nkx protein specifically. The embryos are shown at 36 hpf (A, B, C), 48 hpf (D, E, F), and 72 hpf (G, H, I). The heart phenotype of Nkx2.5-MO embryos was similar to that of control embryos whose ventricle is located at the right side of the atrium when embryos were observed at 36 hpf, 48 hpf and 72 hpf from the ventral view under fluorescence microscope (A, D, G). However, embryos injected with Nkx2.7-MO displayed an unlooping defect from 36 hpf to 72 hpf (B, E, H). Embryos injected with Nkx2.5/2.7-MO displayed a shrunken ventricle and an expanding atrium (C, F, I). v: ventricle; a: atrium.

We designed Nkx2.5- and Nkx2.7-MO to study whether *Nkx2.5* or *Nkx2.7* is required for heart development of zebrafish. Heart-specific-GFP transgenic line Tg (*cmlc2*::GFP) was used to monitor the cardiac morphology in zebrafish embryos. By fluorescence microscope, we observed that embryos injected with 10, 12, and 14 ng of Nkx2.5-MO developed normally from 24 to 72 hpf, although we occasionally observed a small percentage of injected embryos which had a slight degree of pericardial edema. This was also observed in the wild-type embryos and in the embryos injected with a high concentration of control MO (Table 1 and Fig. 1A). In contrast, embryos injected with Nkx2.7-MO at the same concentrations totally failed to complete heart looping during 30 to 72 hpf (Table 1 and Fig. 1B). Moreover, double knockdown of *Nkx2.5* and *Nkx2.7* (Nkx2.5/2.7-MO) by injection of 8 ng Nkx2.5-MO combined with 8 ng of Nkx2.7-MO into embryos displayed a shrunken ventricle and an expanded atrium with an incomplete heart looping. After 72 hpf, many symptoms of heart defects appeared in both Nkx2.7 and Nkx2.5/2.7 morphants, including regurgitation of blood, arrhythmia, string-like heart and pericardial edema (data not shown). The rates of defective heart occurrence in the Nkx2.7 and the Nkx2.5/2.7 morphants were dose-dependent (Table 1). Comparing the cardiac defects among Nkx2.5 morphants, Nkx2.7 morphants and Nkx2.5/2.7 morphants, we observed that the Nkx2.5 morphants did not have any obvious heart defects, while the Nkx2.7 morphants exhibited an unlooping defect with a low percentage of shrunken ventricles. The Nkx2.5/2.7 morphants displayed not only the unlooping defect, but also shrunken ventricles (Table 1).

**Table 1:** The cardiac phenotypes of embryos injected with Nkx2.5-MO, Nkx2.7- MO and Nkx2.5/2.7-MO.

Morpholino (ng)		Survival numbers of Embryos	Abnormal Phenotypes		
Nkx2.5	Nkx2.7		Looping Defects	Shrinking Ventricle	pericardial edema
4	4	142/167 (85.0%)	85(59.8%)	75(52.8%)	—
6	6	207/255 (81.2%)	166 (80.2%)	151(72.9%)	—
8	8	183/233 (78.6%)	177 (97.8%)	162 (88.5%)	—
0	10	115/137 (83.9%)	56(48.7%)	12(10.4%)	—
0	12	139/177 (78.5%)	87(62.5%)	27(19.4%)	—
0	14	183/246 (74.3%)	143(78.1%)	75(40.9%)	—
10	0	24/188 (87.2%)	0	0	21/164(12.8%)
12	0	74/84(88.1%)	0	0	15/76 (19.7%)
14	0	82/98(83.7%)	0	0	19/82(23.2%)

Embryos derived from the transgenic line of Tg(*cmlc2*::GFP) were used, and the cardiac morphology was observed by fluorescence microscope. The major cardiac phenotype of Nkx2.5/2.7-MO morphants revealed unlooping defects and shrunken ventricle; the major phenotype for Nkx2.7-MO morphants was looping defects with a lesser rate of shrunken ventricle. The Nkx2.5-MO morphants displayed no obvious cardiac phenotype except pericardial edema. (“—“ represents no observation.) The percentage of each abnormal phenotype was counted individually.

To confirm that the phenotypes of morphants were specifically induced by the absence of Nkx2.5/2.7 function, we constructed plasmids of pCS2-Nkx2.5-GFP and pCS2-Nkx2.7-GFP, in which the binding sequence of MO was fused with the GFP cDNA. Unlike the GFP was expressed in the embryos (*n* = 173) injected 6 ng Nkx-specific MO together with 150 pg *GFP* mRNA, the GFP expression was not observed in the embryos (n = 155) injected 6 ng Nkx-specific MO together with 150 pg mRNA synthesized either from pCS2-Nkx2.5-GFP or from pCS2-Nkx2.7-GFP (data not shown). Embryos injected either with *Nkx2.5-GFP* and Nkx2.7-MO, or with *Nkx2.7-GFP* and Nkx2.5-MO, distinctively express GFP (data not shown). This evidence indicated that injection of Nkx2.5-MO and Nkx2.7-MO can specifically block the translation of the *Nkx2.5* or *Nkx2.7* mRNA, respectively. We also designed a 5-bp mismatched binding sequence for Nkx2.7 MO, termed Nkx2.7-MO-control, to serve as a control. Results showed that the Nkx2.7-MO-control-injected zebrafish embryos exhibited the wild-type-like phenotype when this control MO was injected at a range of 6.0 to 16 ng. Thus, we conclude that the defective phenotypes of morphants are induced specifically by the injection of Nkx2.5/2.7-MO.

### *Nkx2.5* and *Nkx2.7* function redundantly in zebrafish heart development

To demonstrate the functions shared by *Nkx2.5* and *Nkx2.7*, as well as the specificity of MO for *Nkx2.5* and *Nkx2.7*, the *Nkx2.5* mRNA or the *Nkx2.7* mRNA was co-injected with MO into zebrafish embryos. Results showed that co-injection of 9 ng Nkx2.7-MO with 10 and 20 pg of *Nkx2.5* mRNA in embryos decreased the occurrence of heart defects from 55% to 32% and 22%, respectively (Table 2), indicating that the defective phenotype of Nkx2.7 morphants was specifically rescued by *Nkx2.5* mRNA. Co-injection of 8 ng Nkx2.5-MO plus 8 ng Nkx2.7-MO with 25 and 50 pg of *Nkx2.5* mRNA in embryos decreased the occurrence of heart defects from 91% to 73% and 58%, respectively (Table 2). Furthermore, co-injection of 8 ng Nkx2.5-MO plus 8 ng Nkx2.7-MO with 0.25 and 0.5 pg of *Nkx2.7* mRNA in embryos decreased the occurrence of heart defects from 92% to 61% and to 43%, respectively (Table 2), indicating that the heart defects in embryos were rescued specifically by *Nkx2.5*- and *Nkx2.7*- mRNA. Taken together, the decrease of heart defects across samples indicates that *Nkx2.7* and *Nkx2.5* function redundantly during the heart development of zebrafish embryos. However, since rescue experiments were performed by substantially decreasing the concentration of *Nkx2.7* mRNA, we can conclude that *Nkx2.7* plays a functionally more predominant role in heart formation.

**Table 2:** Functional redundant activities between Nkx2.5 and Nkx2.7 in zebrafish heart development.

Morpholino (ng)		mRNA (pg)		Total (n)	Heart Defects (%)
Nkx2.5	Nkx2.7	*Nkx2.5*	*Nkx2.7*		
0	9 ng	0	0	177	55%
0	9 ng	10 pg	0	109	32%
0	9 ng	20 pg	0	113	22%
8 ng	8 ng	0	0	146	91%
8 ng	8 ng	25 pg	0	278	73%
8 ng	8 ng	50 pg	0	169	58%
8 ng	8 ng	0	0	166	92%
8 ng	8 ng	0	0.25 pg	189	61%
8 ng	8 ng	0	0.5 pg	206	43%

For rescue study, the *Nkx2.5* mRNA was co-injected with Nkx2.7-MO into embryos derived from zebrafish transgenic line Tg(*cmlc2*::GFP). The percentages of heart defects were decreased compared to phenotypes which occurred in the embryos injected with Nkx2.7-MO alone. Similarly, either the *Nkx2.5* or *Nkx2.7* mRNA enabled embryos to be rescued from the defects as a result of the injection of Nkx2.5/2.7-MO. n: total number of embryos analyzed.

### *Nkx2.5* was overexpressed in the Nkx2.7-MO-injected embryos

**Figure 2:**
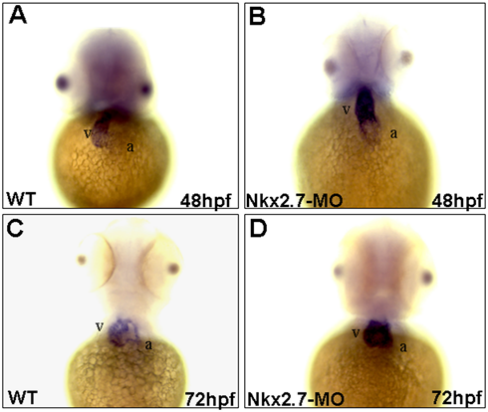
Nkx2.5 was overexpressed in the Nkx2.7-knockdown embryos. Nine nanograms of Nkx2.7-MO were injected into one-cell stage of embryos. *Nkx2.5* was expressed predominantly in ventricle (v) and weakly in atrium (a) of wild-type (WT) embryos at 48 hpf (A), but only minimally and weakly in ventricle at 72 hpf (C). However, the Nkx2.7-MO-injected embryos retained robust *Nkx2.5* expression in ventricle both at 48 and 72 hpf (B and D). The embryonic stages were as indicated, and embryos were observed ventrally. v: ventricle; a: atrium.

In the wild-type embryos, *Nkx2.5* was expressed predominantly in the ventricle and weakly in the atrium at 48 hpf (Fig. 2A), limiting ventricle expression when embryos developed at 72 hpf (Fig. 2C). Unlike the *Nkx2.5* expression in the wild-type, *Nkx2.7*-knockdown morphants retained robust *Nkx2.5* expression in ventricle (Figs. 2B and 2D). Because *Nkx2.7* was knocked down and *Nkx2.5* was overexpressed, these results also support the hypothesis that Nkx2.5 and Nkx2.7 play similar roles in cardiac morphogenesis in zebrafish and that, moreover, in the absence of one, the other can compensate for the loss of function.

### The ventricle became smaller and consisted of a single layer in the Nkx2.5 and Nkx2.7 double knockdown embryos

**Figure 3:**
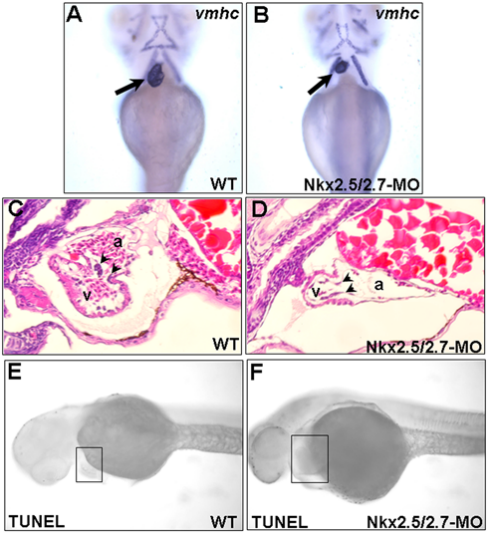
Ventricle becomes smaller and consists of a single layer in the Nkx2.5 and Nkx2.7 double knockdown morphants (Nkx2.5/2.7-MO). Ventricular myosin heavy chain (*vmhc*) was used as a probe to detect the ventricle morphology in (A) wild-type (WT) and (B) Nkx2.5/2.7-MO embryos at 72 hpf. The ventricle of Nkx2.5/2.7-MO embryos was smaller than that of WT embryos (indicated by arrows). Hematoxylin and eosin staining showed that ventricular myocardium of WT was two or more cell layers in thickness. However, only one cell layer was retained in the ventricular myocardium of the Nkx2.5/2.7-MO embryos. In addition, compared to wild-type embryos, the endocardium of Nkx2.5/2.7-MO-injected embryos did not form endocardial cushion (indicated by arrowheads in C and D). Like WT embryos, TUNEL assay did not display the increase of TUNEL-positive cells in the heart region of Nkx2.5/2.7-MO embryos at 40 hpf (indicated by boxes in E and F). Embryos were observed ventrally (A–B) or laterally (C–F). v: ventricle; a: atrium.

In addition to monitoring cardiac morphology by using heart-tagged GFP zebrafish line Tg(*cmlc2*::GFP), we used *vmhc* as a ventricle-specific probe to determine the ventricular cell fate. Compared to the wild-type, the ventricle of the *Nkx2.5* and *Nkx2.7* double knockdown embryos became smaller when they were observed at 72 hpf. However, the expression level of *vmhc* in the heart of the Nkx2.5/2.7- MO-injected embryos was similar to that of wild type (Figs. 3A and 3B). It is worth noting that neither WT nor Nkx2.5/2.7 morphant embryos expressed *vmhc* in the head muscle until 72 hpf. Therefore, the Nkx2.5/2.7-MO morphants did not appear less developed than WT embryos. This evidence demonstrates that, although the ventricular fate is properly determined in the Nkx2.5/2.7-MO-injected embryos, their ventricular morphogenesis is abnormal.

In order to understand why the ventricle of Nkx2.5/2.7-MO-injected embryos is smaller than that of wild-type embryos, we performed a histological examination of the 72-hpf embryos. Compared to the wild-type (Fig. 3C), a thinning layer of myocardium with fewer cell numbers in the ventricle was observed in the Nkx2.5/2.7 morphants (Fig. 3D). Additionally, the endocardium was not observed to form an endocardial cushion in these morphants (Fig. 3D). Finally, the TUNEL assay demonstrated that the decrease of myocardium cell numbers in the ventricle did not result from cell death, but rather from defective proliferation of ventricular myocardium, which led to a smaller heart with a thinning layer (Figs. 3E and 3F).

### Heart deterioration occurring in the Nkx2.5/2.7 morphants results from defective myocardial differentiation

**Figure 4:**
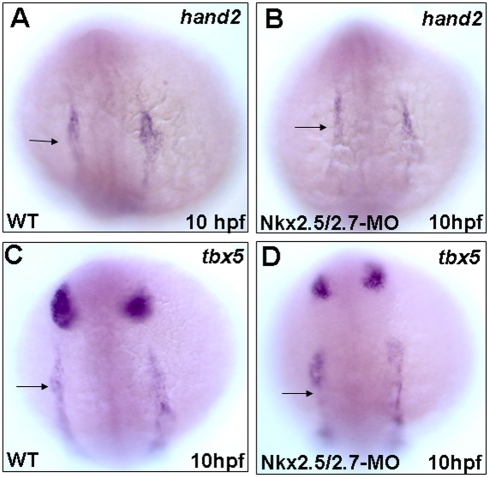
Early cardiac markers express normally in the Nkx2.5 and Nkx2.7 double knockdown morphants (Nkx2.5/2.7-MO). Whole mount *in situ* hybridization showed that the expression patterns of *hand2* and *tbx5*, lateral plate mesoderm markers, were similar between wild-type (WT) and Nkx2.5/2.7-MO embryos at 10 hpf (A vs. B; C vs. D, respectively). Arrows: heart field.

The expressions of *tbx5* and *hand2* in the cardiac precursor regions were normal in the Nkx2.5/2.7-MO-injected embryos at 12 hpf (Figs. 4A and 4C vs. 4B and 4D). These two markers were present in the bilateral regions of the lateral plate mesoderm and showed the correct anterioposterior localization with the same expression level. Thus, the early cardiac marker genes were transcribed normally in the Nkx2.5/2.7 morphants.

**Figure 5:**
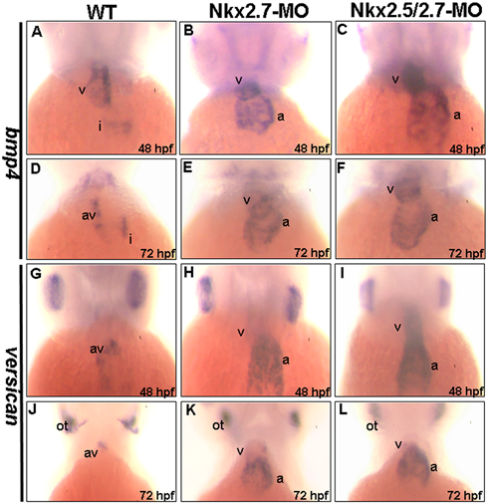
Abnormal cardiac differentiation occurred in the Nkx2.7-knockdown zebrafish embryos. The expressions of *bmp4* (A–F) and *versican* (G–L) in hearts were compared between wild-type (WT) (A, D, G, J), Nkx2.7-MO- (B, E, H, K) and Nkx2.5/2.7-MO- injected embryos (C, F, I, L) at 48 (A–C, G–I) and 72 hpf (D–F, J–L). In WT embryos, *bmp4* was expressed in the ventricle and inflow tract at 48 hpf (A), and then *bmp4* was restricted in its expression at the AV boundary at 72 hpf (D). However, in the Nkx2.7-MO (B, E) and Nkx2.5/2.7-MO (C, F) embryos, *bmp4* was still expressed predominantly in the ventricle and atrium from 48 to 72 hpf. Similarly, in WT embryos, the *versican* expression was more predominant in ventricle than in atrium, at about 31 to 33 hpf, and then *versican* was confined in its expression at the AV boundary after 33 hpf (G, J). In contrast, in the Nkx2.7-MO (H, K) and Nkx2.5/2.7-MO (I, L) embryos, the *versican* was significantly expressed in the atrium and ventricle. In addition, the *versican* expression pattern in otoliths remained unchanged (J, K, and L). All images are ventral views, anterior to the top. a: atrium; v: ventricle; i: inflow tract; av: atrioventricular boundary; ot: otoliths.

We also observed myocardial differentiation markers, such as *bmp4*, *versican*, *tbx5*, and *tbx20*. BMP4 is one of the TGF-β superfamily proteins and is involved in valve development during the cardiac maturation stage. A ventricle-enriched expression of *bmp4* was the same between wild-type and Nkx-deficient embryos from 31 to 33 hpf (data not shown). Moreover, in wild-type embryos, *bmp4* was found to express in the ventricle and inflow tract at 48 hpf (Fig. 5A), and then it was expressed exclusively in the atrioventricular junction at 72 hpf (Fig. 5D). However, unlike the dynamic expression of *bmp4* in the wild-type embryos, *bmp4* in the Nkx2.7 and in the Nkx2.5/2.7 morphants failed to change its predominant ventricular and atrial expression pattern (Figs. 5B and 5E; 5C and 5F). Similarly, *versican* was expressed broadly in the atrium and weakly in the ventricle of the wild-type embryos at 33 hpf. This atrium-enriched expression of *versican* was the same between wild-type and Nkx-deficient embryos (data not shown). After 36 hpf, the *versican* expression of wild-type was restricted to the AV boundary (Figs. 5G and 5J). However, Nkx2.7 and the Nkx2.5/2.7 morphants still expressed a high level *versican* in the atrium of the heart from 48 to 72 hpf (Figs. 5H and 5K; 5I and 5L). Nevertheless, we noticed that the *versican* was expressed in otoliths of both the wild-type embryos and the Nkx-deficient embryos by 72 hpf (Figs. 5J, 5K and 5L), indicating that the development of Nkx-deficient embryos was not delayed and that the defects caused by Nkx-MO were specific in the heart, not in otoliths (Figs. 5J, 5K and 5L).

**Figure 6:**
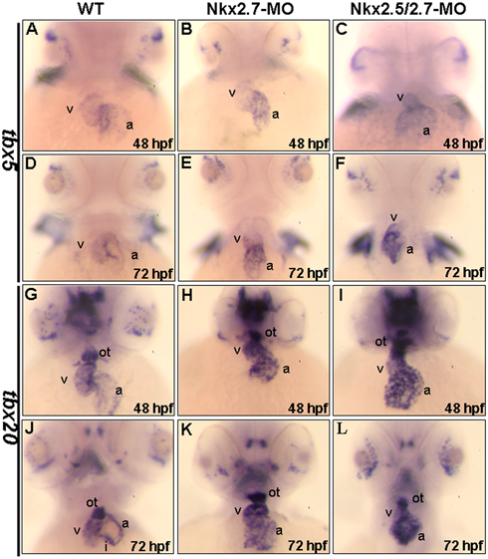
Chamber maturation was affected in the Nkx2.7-knockdown zebrafish embryos. The expressions of *tbx5* (A–F) and *tbx20* (G–L) in hearts were compared between wild-type (WT) (A, D, G, J), Nkx2.7-MO- (B, E, H, K) and Nkx2.5/2.7-MO- injected embryos (C, F, I, L) at 48 (A–C, G–I) and 72 hpf (D–F, J–L). In WT embryos, *tbx5* was expressed strongly in ventricle, but weakly in atrium at 48 hpf and beyond (A, D). However, in the Nkx2.7-MO (B, E) and Nkx2.5/2.7-MO (C, F) embryos, *tbx5* retained its strong expression in the heart, although the expression pattern was gradually changed from ventricle-enriched expression to atrium-enriched expression from 48 hpf to 72 hpf (B and E; C and F). In WT embryos, *tbx20* expression was similar to the expression gradient of *tbx5* in the heart at 48 hpf, and the *tbx20* expression was stronger than that of *tbx5*. In addition, *tbx20* expression was also detected in outflow tract at 48 hpf (G), and *tbx20* expression was restricted to outflow tract and inflow tract by 72 hpf (J). However, in Nkx2.7-MO (H, K) and Nkx2.5/2.7-MO (I, L) embryos, *tbx20* was expressed strongly in the heart and outflow tract from 48 to 72 hpf. All images are ventral views, anterior to the top. v: ventricle; a: atrium. ot: outflow tract; i: inflow tract.

In the wild-type embryos, *tbx5* expression was slightly greater in the atrium than the ventricle at 26 hpf, which was consistent with what Garrity (2002) reported [25]. Then, the gradient expression of *tbx5* was changed from atrium-rich expression to ventricle-rich expression at 48 hpf (Figs. 6A and 6D). However, in the Nkx2.7 and Nkx2.5/2.7 morphants, the *tbx5* expression remained atrium-rich at 48 hpf (Figs. 6B and 6C), and even beyond 48 hpf (Figs. 6E and 6F). In an overview of the whole heart, the expression level of *tbx5* was greater in morphants than the wild-type embryos. Although the dynamic change of *tbx5* expression pattern from atrium to ventricle in the wild-type at later stages did not occur in the Nkx2.7 or the Nkx2.5/2.7 morphants, the expression level of *tbx5* in pectoral fin bud of morphants was the same as wild-type embryos.

We also detected another myocardial differentiation marker, *tbx20*, which was expressed during cardiac development. In the wild-type, this marker was expressed more predominantly in the ventricle than in the atrium at 48 hpf (Fig. 6G). The expression of *tbx20* was confined to the outflow tract, AV canal and inflow tract at 72 hpf (Fig. 6J); however, its expression in the Nkx2.7 and the Nkx2.5/2.7 morphants maintained a strong signal in the ventricle and atrium of the heart (Figs. 6H and 6K; 6I and 6L). While this behavior was unlike the *tbx20* expression patterns in the wild-type embryos, the expression level of *tbx20* in the outflow tract of morphants was, nevertheless, the same as wild-type embryos. Still, we noticed that, like wild-type embryos, the expression level of *tbx20* in tegmentum of the Nkx2.7 and Nkx2.5/2.7 morphants started to gradually decrease from 48 to 72 hpf (Figs. 6J, 6K and 6L), indicating that, although all these morphants developed to later stages without delay, they also exhibited specific heart defects.

In summary, we examined the expression patterns of myocardial differentiation markers, including *bmp4*, *versican*, *tbx5* and *tbx20*, in the Nkx2.7-MO- and Nkx2.5-/2.7-MO-injected embryos. While the expression levels of these markers were the same as those at the early stage of the wild-type, we found that they became more intensive than those of the wild-type at the later stage, suggesting that myocardial differentiation is defective in the morphants. We also noticed that the hearts of these Nkx2.7- and Nkx2.5/2.7-MO-injected embryos finally deteriorated and became string-like in form without undergoing further development. Taken together, this line of evidence suggests that the late onset of myocardial differentiation results in heart defects at a later maturation stage of cardiogenesis in the Nkx-knockdown embryos.

### Overexpression of *Nkx2.7* or *Nkx2.5* causes cardiac defects similar to those induced by Nkx-knockdown

**Figure 7:**
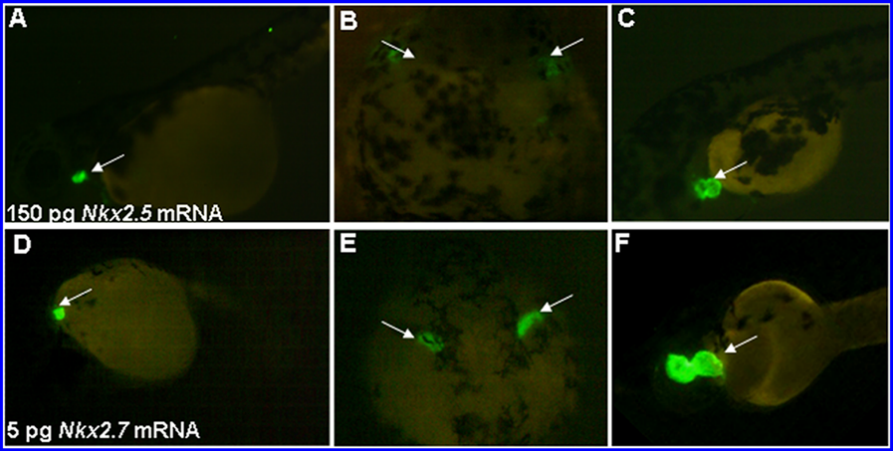
Overexpression of *Nkx2.5* and *Nkx2.7* in embryos resulted in serious heart defects. Amounts equaling 150 pg *Nkx2.5* mRNA (A–C) and 5 pg *Nkx2.7* mRNA (D–F) were injected individually into one-celled stage embryos derived from transgenic line Tg(*cmlc2*::GFP), whose hearts were specifically tagged with green fluorescent protein. We observed that there were many phenotypes of heart defects at 48 hpf resulting from the overexpression of either *Nkx2.5* mRNA or *Nkx2.7* mRNA. These defects included malposition of the reducing heart (A, D), bilateral heart (B, E), small heart (C) and large heart (F). Interestingly, overexpression by injection of *Nkx2.7* mRNA as low as 5 pg caused an effect similar to that induced by injection 30 greater times than that of *Nkx2.5* mRNA. (A, C, D, F), lateral views; (B, E), dorsal views; Arrows: green fluorescent heart.

To examine whether *Nkx2.7* plays roles similar to those of *Nkx2.5* in cardiogenesis, we studied the effect of Nkx overexpression on heart development by injection of either *Nkx2.7* or *Nkx2.5* mRNA into one-celled stage embryos derived from transgenic line Tg(*cmlc2*::GFP), which has heart-specific GFP. By fluorescence microscopy, we observed that overexpression of 5 pg *Nkx2.7* mRNA caused defective phenotypes similar to overexpression of 150 pg *Nkx2.5* mRNA. These defects included a small patch of cardiac cells (64% in the 150-pg-*Nkx2.5*-overexpressed embryos; 28% in the 5-pg-*Nkx2.7*-overexpressed embryos), malposition of cardiac migration (13% in the 150-pg-*Nkx2.5*-overexpressed embryos; 2% in the 5-pg- *Nkx2.7*-overexpressed embryos), and an enlarged heart (18% in the 5-pg- *Nkx2.7*-overexpressed embryos) (Figs. 7A–F). Moreover, injection of *Nkx2.5* mRNA in the amount of 150 pg also caused 41% of embryos (62/150) to have a dorsoventral axial defect, whereas injection of *Nkx2.5* mRNA in the amount of 50 pg caused 22% of embryos (30 out of 135) to have an enlarged heart (data not shown). Based on this evidence, we suggest that *Nkx2.7* carries out functions similar to those of *Nkx2.5*, but that *Nkx2.7* appears to play a more prominent role in zebrafish heart development. This conclusion is based on the contrasting concentrations of mRNA required to generate similar heart defects; i.e., 5 pg *Nkx2.7* mRNA vs. 150 pg *Nkx2.5* mRNA.

### The expressions of *tbx5* and *tbx20* are modulated by *Nkx2.7*

**Figure 8:**
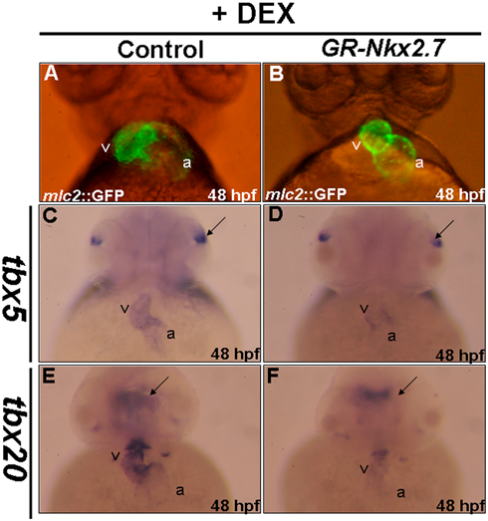
The expressions of *tbx5* and *tbx20* were modulated by *Nkx2.7*. Each embryo derived from transgenic line Tg(*cmlc2*::GFP), whose heart was specifically tagged with green fluorescent protein, was injected with 0.15 ng of plasmid, in which *Nkx2.7* mRNA was transcribed conditionally by adding dexamethasone. All embryos were treated with dexamethasone at 10 hpf and took a ventral view at 48 hpf to observe the cardiac development under the fluorescence microscope. The cardiac morphologies of the control embryos and the *GR-Nkx2.7*-overexpression embryos were shown. Compared to the control (A), various cardiac defects were found in around 35% of embryos from the *GR-Nkx2.7*-overexpression group, including unlooping and shrunken heart (B). In addition, the expressions of *tbx5* (C, D) and *tbx20* (E, F) were down-regulated in the *GR-Nkx2.7*-overexpresion embryos. However, the expression levels of *tbx5* in eyes (indicated by arrows in C and D) and *tbx20* in brain (indicated by arrows in E and F) remained unchanged. v: ventricle; a: atrium.

In addition to the cardiac defects occurring in the embryos that were overexpressed in either *Nkx2.5* or *Nkx2.7*, we noticed that embryos injected with 150 pg of either *Nkx2.5* or *Nkx2.7* mRNA suffered a severe defect in ventralization (data not shown). However, this ventralization defect was not observed in the embryos that were treated with either Nkx2.7- or/and Nkx2.5-knockdown. This inconsistency between loss- and gain-of-function of Nkx2.5 or Nkx2.7 may result from the possibility that either Nkx2.7 or Nkx2.5 plays additional roles in zebrafish heart development before the late-gastrula period. Therefore, in order to further confirm the specific effects caused by the overexpression of *Nkx2.7* in the heart after gastrulation, we constructed a dexamethasone-inducible plasmid in which the ligand-binding domain of GR was fused with the coding region of *Nkx2.7*. When we added dexamethasone to the 10-hpf embryos derived from the transgenic line Tg(*cmlc2*::GFP), these embryos were fixed at 48-hpf. Results showed that the cardiac maturation was defective, including an unlooping or shrunken heart (Fig. 8B), although the dorsoventral axis formation was not affected in the GR-Nkx2.7-produced embryos (Figs. 8D and 8F). We also noticed that, unlike wild-type embryos, the expressions of *tbx5* and *tbx20* in these GR-Nkx2.7-produced embryos were reduced at 48 hpf (Figs. 8D and 8F). Moreover, compared to the unchanged expression levels of *tbx5* in eyes (Fig. 8C vs. 8D) and *tbx20* in brain (Fig. 8E vs. 8F), the effect of *Nkx2.7* overexpression on the reduced expressions of *tbx5* and *tbx20* was heart specific. This evidence strongly suggests that Nkx2.7 modulates the expression of *tbx5* and *tbx20*, which, in turn, affects cardiac differentiation.

## Discussion

Using two Nkx2.5- and Nkx2.7-specific MOs, we demonstrated that *Nkx2.5* and *Nkx2.7* are required for normal heart development. Prior to the onset of cardiac looping, cardiogenesis progresses normally in both Nkx2.7-MO and Nkx2.5/2.7-MO, indicating that Nkx is not an essential requirement for the early stage of heart development. However, at 36 hpf, when heart development reaches the looping stage, *Nkx2.7* morphants showed an unlooping defect. Moreover, *Nkx2.5/2.7* morphants not only displayed an unlooping defect, but also a shrunken ventricle. In addition, the expression of differentiated marker genes, including *bmp4*, *versican*, *tbx5* and *tbx20*, was abnormal either in the ventricle or in both the ventricle and atrium (Figs. 5 and 6). Although the formation of two distinct chambers was enabled in *Nkx2.7* and *Nkx2.5/2.7* morphants, the hearts were not processed further, remaining, instead, a linear tube, resulting in abnormal contractions and arrhythmia.

*Nkx2.7*, a member of the *tinman* family of related genes, contains the *tinman*-like amino terminal decapeptide, homeobox, and NK2 domain. *Nkx2.7* exhibits temporal and spatial expression patterns very similar to *Nkx2.5* during zebrafish cardiac development, although the onset of *Nkx2.7* at the 10.5 hpf comes somewhat earlier than that of *Nkx2.5*. In addition, *Nkx2.7* is also detected with a co-localized expression pattern at pharyngeal endoderm progenitors like *Nkx2.3* [19]. These expression profiles indicate that *Nkx2.7* and *Nkx2.5* are closely associated in the roles they play in the context of the regulation of cardiac differentiation.

In *Drosophila*, *tinman* is essential for primary cell lineage determination and early morphogenesis. Embryos that lack *tinman* function do not develop any dorsal vessel or gut muscle progenitor cells [6]. *Nkx2.5*, the mouse homolog of *tinman*, is an early marker of cardiomyocyte precursors. In the knockout of *Nkx2.5* by gene targeting techniques, mouse embryos are arrested at the looping stage [7]. Surprisingly, this mouse *Nkx2.5*-knockout embryo does not exhibit the same defective phenotype of *tinman* null embryos, indicating that there are other *Nkx2.5* homologs to compensate for *Nkx2.5* loss of function in mouse heart development. For example, Nkx2.6 compensates for Nkx2.5 functions in the pharyngeal endoderm of mouse embryos, but not in hearts [14]. Recently, mouse Nkx2.7 was found (GeneID: 108060). Thus, it is worthwhile to study whether mouse Nkx2.7 functions redundantly with mouse Nkx2.5 in a way that is similar to zebrafish *Nkx2.7* and *Nkx2.5*. In *Xenopus*, Cleaver *et al.* (1996) [9] suggested that *XNkx2.3* and *XNkx2.5* play the same roles in cardiac development. In zebrafish, we demonstrate in this study that *Nkx2.7* does compensate for the loss of *Nkx2.5* function in cardiac morphogenesis.

Recently, Targoff *et al.* (2008) [26] demonstrated that Nkx2.5/Nkx2.7 morphants of zebrafish are well developed at early embryonic stage; however, morphants fail to elongate normally at heart tube stage. Thus, the authors concluded that abnormal heart tube extension will cause cardiac differentiation effects on ventricular and atrial cell numbers. In this study, we also observed similar phenotypes of Nkx2.5/Nkx2.7 morphants in that the heart tubes of Nkx2.5/Nkx2.7 morphants cannot extend further and the ventricle portion displays shorter and wider than that of wild-type embryo. However, in our study, we described the defective phenotypes and studied the functions of Nkx2.5/Nkx2.7 in more detail. To summarize, we first observed the cardiac phenotype at various developmental stages, including the period from 26 to 55 hpf when zebrafish ventricle is undergoing differentiation and proliferation. Targoff *et al.* (2008) [26], on the other hand, did not investigate this important period of time. Second, by taking the advantage of zebrafish transgenic line Tg(*cmlc2*::GFP), which possesses the heart-specific GFP signal, we were able to observe the dynamic change of ventricular morphology in live embryos. In so doing, we found that the ventricle of Nkx2.5/Nkx2.7 morphants retained the pre-mature phase during this period rather than undergoing further cardiac morphogenesis. Thus, we suggested that the differentiation and proliferation of ventricle in Nkx2.5/Nkx2.7 morphants may cease after 26 hpf, resulting in the decrease of cell numbers of ventricle at the later stages. Third, by detecting the differentiated markers, such as *versican*, *bmp4*, *tbx5* and *tbx20*, we also found that the expression patterns of these markers in Nkx-deficient embryos were the same as those of wild type at early heart tube stage. However, the expressions of these markers in Nkx-deficient embryos were not restrained to the places where they should be confined at later cardiac differentiation stage. Based on these lines of evidence, we suggest that the decrease of ventricular myocardial proliferation and the defective myocardial differentiation both result from the up-regulation of *bmp4*, *versican*, *tbx5* and *tbx20* at late stage, even though these genes are all expressed normally in hearts at an early heart tube stage. Fourth, we performed a histological examination of the 72-hpf embryos and found that, compared to the wild-type, a thinning layer of myocardium with fewer cell numbers in the ventricle was observed in the Nkx2.5/2.7 morphants and that the endocardium of morphants did not form an endocardial cushion (Figs. 3C and 3D). Fifth, by using histological section of the Nkx2.5/2.7-MO-injected embryos, we found that the formation of atrioventricular valves was abnormal at 72 hpf (Fig. 3D).

We observed that the heart of Nkx2.5/2.7-MO double-knockdown morphants appeared to have a vigorous and rhythmic peristaltic contraction initially. Subsequently, however, the hearts of these double knockdown morphants failed to undergo looping morphogenesis, and the contractile function diminished progressively. Moreover, blood was regurgitated within the heart chamber after 72 hpf, and the heart became silent with cessation of blood circulation by 84 hpf. These defective phenotypes could have resulted from valve dysfunction. Interestingly, both *bmp4* and *versican* are restricted to atrioventricular boundary of heart at the later stage of heart development [27]. Both genes are involved in cardiac valve formation and both are examined in this study. Moreover, it has been found that mutation of *Apc* [28] or *NXT2* [29] of zebrafish showed that expression of *bmp4* and *versican* genes are upregulated and that domains are expanded throughout the hearts. Similarly, in this study, we found that *bmp4* and *versican* are dramatically upregulated and that the domains of expression are greatly expanded to the ventricle in the hearts of Nkx2.5/2.7 morphants. Therefore, we speculate that blood regurgitation occurring in the Nkx2.5/2.7 morphants' hearts resulted from valve dysfunction, which, in turn, causes the defective morphogenesis and function of heart.

Prall *et al.* (2007) [30] used cDNA microarray analysis to compare the transcripts of mouse Nkx2.5 heterozygous and null mutant embryos from E8.0 to E9.5 and found that *tbx5* is upregulated two-fold greater in null embryos at E8.0-E8.5. We found that both Nkx2.7 and Nkx2.5/2.7 morphants exhibit overexpression of *tbx5* and *tbx20* in heart, suggesting that Nkx2.5 and Nkx2.7 may be negative modulators of *tbx5* and *tbx20* in zebrafish. Therefore, the finding of Prall *et al.* supports our present study. On the other hand, Prall *et al.* (2007) [30] also demonstrated that there is an Nkx2.5/Bmp2/Smad1 negative loop pathway to regulate heart precursor specification and proliferation. They pointed out that *Nkx2.5* null mutants cause over-specification of cardiac progenitors and that the area of over-specification of cardiac progenitors appears to be a broad expression of early cardiac markers in the cardiac crescents. Most importantly, Prall *et al.* (2007) [30] further stated that impaired proliferation of secondary heart field-derived cells, which contribute to most ventricle cells, results from over-specification of cardiac progenitors. For this reason, *Nkx2.5* null mutants have a thin ventricular myocardium.

However, in our study, the expression of early cardiac markers, *tbx*5 and *hand*2, do not exhibit expansion of cardiac progenitors (Figs. 4A–4D), indicating that the over-specification of cardiac progenitors does not occur. Instead, the Nkx2.5/2.7-MO-injected embryos consist of a single layer of ventricle, which does not result from over-specification of progenitors in a manner similar to mouse *Nkx2.5* null mutants. At the same time, *tbx5* was overexpressed in the ventricle and atrium of zebrafish (Fig. 6F). The fact that overexpression of *tbx5* in the heart inhibits cardiomyocyte proliferation has been demonstrated in mice [31] and chicks [32], suggesting that the function of *tbx5* acts as growth arrest signal to regulate cellular proliferation. Thus, it is reasonable to conclude that the thin-layer of heart of zebrafish *Nkx2.5/2.7* morphants may result from the effect of higher expression of *tbx5*. Therefore, we reason that the heart defect induced by the knockdown of *Nkx2.5/2.7* is more likely to be caused by the late onset of cardiac differentiation. It was also demonstrated in this study that *Nkx2.7* is a modulator of *tbx5* and *tbx20* expression, which further supports the conclusion noted above and gives added support to the prominent role *Nkx2.7* plays in cardiac morphogenesis.

Chen and Fishman (1996) [10] reported that overexpression of *Nkx2.5* (100 pg) in zebrafish causes a large hyperplastic heart with normal function and chamber morphology. Moreover, injection of a higher dose (250 pg) of *Nkx2.5* mRNA affected the dorsoventral axis of embryos severely. Cleaver *et al.* (1996) [9] also demonstrated that overexpression of *Nkx2.5* and *Nkx2.3* in *Xenopus* resulted in a hyperplastic heart. In our study, injection of 150 pg of *Nkx2.5* mRNA or 5 pg of *Nkx2.7* mRNA obtained results similar to Chen and Fishman (1996) [10]. Injection of 50 pg of *Nkx2.5* mRNA caused 22% of embryos (30 out of 135; 30/135) to have an enlarged heart and injection of 150 pg of *Nkx2.5* mRNA caused 64% of embryos (67/104) to have a reduced heart, whereas injection of 5 pg of *Nkx2.7* mRNA caused 18% (32/182) and 28% (51/182) of embryos to have a reduced heart and an enlarged heart, respectively. Our data are supported by the paper published by Chen *et al.* (1996) [10], who demonstrated that embryos injected with 100 pg *Nkx2.5* mRNA showed an enlarged heart, but embryos injected with 250 pg *Nkx2.5* mRNA displayed bilateral beating heart, reduced heart or dorsal-ventral axial defects. We speculate that ectopic expression of *Nkx2.5* mRNA at the one-cell stage results in nonspecific defects in zebrafish embryos. For example, there are no reports about Nkx2.5 function in dorsal-ventral axis formation. Based on this point, we suggest that *Nkx2.7* carries out functions similar to those of *Nkx2.5*, but, because there may be many unexpected effects on embryos from overexpression of either *Nkx2.5* or *Nkx2.7*, we performed a specific knockdown of morpholino and a GR-inducible assay to learn the exact functions of Nkx2.5 and Nkx2.7 in zebrafish development. Specifically, we note that injection of 5 pg *Nkx2.7* mRNA results in a phenotype similar in defect to the injection of 150 pg *Nkx2.5* mRNA (Fig. 7). Compared to the injection dosage of *Nkx2.5* mRNA, we notice that only a one-thirtieth concentration of *Nkx2.7* mRNA is required to rescue the defects induced by *Nkx2.5* and *2.7* double knockdowns (Table 2), suggesting that *Nkx2.7* has more effective function than *Nkx2.5*.

Finally, in the loss-of-function experiment, the ventralized embryos were not observed in the Nkx2.5/2.7-knockdown embryos. In contrast, these results were observed in the gain-of-function experiment, in which *Nkx2.5* and *Nkx2.7* were overexpressed. However, based on the GR-Nkx2.7 inducible experiment where *Nkx2.7* is induced after gastrulation, we proved that *Nkx2.7* has a specific effect on heart development, but not axis formation (Fig. 8). Therefore, we speculate that this inconsistency between gain-of-function and loss-of-function experiments may result from the fact that *Nkx2.5* and *Nkx2.7* are not expressed until late-gastrula stage. However, since either the overexpressed *Nkx2.5* or *Nkx2.7* is injected at the one-cell stage, these misexpressional genes can cause abnormal axis formation in embryogenesis. In conclusion, we find that *Nkx2.7* and *Nkx2.5* act as functional homologs of *tinman* in the zebrafish embryos. While *Nkx2.5* and *Nkx2.7* play redundant roles in cardiac morphogenesis, *Nkx2.7* appears to have a more critical function in its effect on cardiac differentiation, as indicated by the gain-of-function and loss-of-function experiments where *Nkx2.7* is observed to regulate the expressions of *tbx5* and *tbx20* through the heart tube stage.

## Materials and Methods

### Fish husbandry and observation

The wild-type AB strain [33] and the transgenic line Tg (*cmlc2*::*GFP*) [34] of zebrafish were cultured at 28.5°C. Embryos were staged by hours post fertilization (hpf) [35]. The phenotype of heart formation was observed under a fluorescent stereomicroscope, MZ FLIII (Leica). Images were captured with a Fine pix S2 pro camera (Nikon) using the Camera Shooting software.

### Morpholino (MO) knockdown

The MOs designed specifically for blocking the translation of *Nkx2.5* and *Nkx2.7* mRNAs were TCATTTGGCTAGAGAACATTGCCAT (Nkx2.5-MO) and GTCACAGGACTCGGAAGCATCGTGC (Nkx2.7-MO), respectively. A control MO specific for *Nkx2.7* was designed as GTgACAcGACTCcGAAGgATCGTcC (Nkx2.7-MO-control), in which a 5-bp mismatched sequence of *Nkx2.7* is indicated in lower case. All MOs were prepared at a stock concentration of 1 mM and diluted to the desired concentration for microinjection into each embryo.

### Plasmid construct

In order to further demonstrate the specificity of MO targeting, we constructed plasmids of pCS2-Nkx2.5-GFP and pCS2-Nkx2.7-GFP, in which the binding sequence of either Nkx2.5-MO or Nkx2.7-MO was fused with the GFP reporter cDNA. The *Not*I-cut pCS2-Nkx2.5-GFP, pCS2-Nkx2.7-GFP or pCS2-GFP served as a template to synthesize RNA using the mMessage Machine kit (Ambion). The synthesized RNA (100 pg per embryo) was co-injected with Nkx-specific MO or alone into one-celled zebrafish embryos. The appearance of GFP in the treated embryos was observed at the 24-hpf stage using green fluorescent microscopy.

In order to avoid nonspecific effect of the overexpressed *Nkx2.7* in the early gastrula stage, we constructed a dexamethasone-inducible plasmid, pCS2GR-Nkx2.7, in which the human glucocorticoid receptor (GR) ligand binding domain was fused with *Nkx2.7*.

### Whole-mount *in situ* hybridization and TUNEL assay

Whole-mount *in situ* hybridization was performed as previously described [36]. Antisense probes used in this study were as follows: *versican* [27]; *bmp4* [37]; *tbx5* [25]; *tbx20* [38] and *vmhc* [39]. The TUNEL assay was performed as described previously [40] using The DeadEnd™ Colorimetric TUNEL System (Promega).

### Rescue and overexpression experiments

Capped mRNA transcripts of *Nkx2.5* and *Nkx2.7* for either rescue or overexpression experiments were synthesized by SP6 *in vitro* transcription according to the protocol of the manufacturer (Epicentre). We synthesized the truncated *Nkx2.5* and *Nkx2.7* mRNAs that did not include the specific MO-target site to avoid affecting rescue efficiency. The resultant mRNAs were diluted to the desired concentration (from 150 to 250 pg/µl), and approximately 2.3 nl was microinjected into one-celled stage embryos. For the GR-induced experiment, capped mRNA of a *NotI*-cut CS2 template contained an insert of the human GR ligand binding domain fused with Nkx2.7. The resultant mRNAs were diluted to 2.3 nl of RNA at a concentration of 0.15 ng and were injected into one-celled embryos. When the injected embryos developed at gastrula stage (10-hpf), we immersed embryos with 10 um dexamethasone in phenylthiourea water to induce the synthesis of GR-Nkx2.7.

### Histology

For paraffin section, embryos were collected at 72 hpf and fixed in 4% PFA for 16 h. Then they were decalcified, dehydrated in a graded series of ethanol, and embedded in paraffin for sectioning into a 5-µm thickness [41]. The resultant sections were rinsed in distilled water, washed with 0.01 M phosphate buffered saline, and stained with hematoxylin and eosin.

## References

[1] Stainier DY (2001). Zebrafish genetics and vertebrate heart formation.. Nat Rev Genet.

[2] Bruneau BG (2002). Transcriptional regulation of vertebrate cardiac morphogenesis.. Circ Res.

[3] Brand T (2003). Heart development: molecular insights into cardiac specification and early morphogenesis.. Dev Biol.

[4] Srivastava D (2006). Making or breaking the heart: from lineage determination to morphogenesis.. Cell.

[5] McGinnis W, Krumlauf R (1992). Homeobox genes and axial patterning.. Cell.

[6] Bodmer R (1993). The gene tinman is required for specification of the heart and visceral muscles in Drosophila.. Development.

[7] Lyons I, Parsons LM, Hartley L, Li R, Andrews JE (1995). Myogenic and morphogenetic defects in the heart tubes of murine embryos lacking the homeo box gene Nkx2-5.. Genes Dev.

[8] Schultheiss TM, Xydas S, Lassar AB (1995). Induction of avian cardiac myogenesis by anterior endoderm.. Development.

[9] Cleaver OB, Patterson KD, Krieg PA (1996). Overexpression of the tinman-related genes XNkx-2.5 and XNkx-2.3 in Xenopus embryos results in myocardial hyperplasia.. Development.

[10] Chen JN, Fishman MC (1996). Zebrafish tinman homolog demarcates the heart field and initiates myocardial differentiation.. Development.

[11] Harvey RP (1996). NK-2 homeobox genes and heart development.. Dev Biol.

[12] Biben C, Hatzistavrou T, Harvey RP (1998). Expression of NK-2 class homeobox gene Nkx2-6 in foregut endoderm and heart.. Mech Dev.

[13] Tanaka M, Yamasaki N, Izumo S (2000). Phenotypic characterization of the murine Nkx2.6 homeobox gene by gene targeting.. Mol Cell Biol.

[14] Tanaka M, Schinke M, Liao HS, Yamasaki N, Izumo S (2001). Nkx2.5 and Nkx2.6, homologs of Drosophila tinman, are required for development of the pharynx.. Mol Cell Biol.

[15] Komuro I, Izumo S (1993). Csx: a murine homeobox- containing gene specifically expressed in the developing heart.. Proc Natl Acad Sci USA.

[16] Lints TJ, Parsons LM, Hartley L, Lyons I, Harvey RP (1993). Nkx-2.5: a novel murine homeobox gene expressed in early heart progenitor cells and their myogenic descendants.. Development.

[17] Evans SM, Yan W, Murillo MP, Ponce J, Papalopulu N (1995). tinman, a Drosophila homeobox gene required for heart and visceral mesoderm specification, may be represented by a family of genes in vertebrates: XNkx-2.3, a second vertebrate homologue of tinman.. Development.

[18] Buchberger A, Pabst O, Brand T, Seidl K, Arnold HH (1996). Chick Nkx2-3 represents a novel family member of vertebrate homologues to the Drosophila homeobox gene tinman: differential expression of cNKx-2.3 and cNkx 2-5 during heart and gut development.. Mech Dev.

[19] Lee KH, Xu Q, Breitbart RE (1996). A new tinman-related gene, nkx2.7, anticipates the expression of nkx2.5 and nkx2.3 in zebrafish heart and pharyngeal endoderm.. Dev Biol.

[20] Pabst O, Schneider A, Brand T, Arnold HH (1997). The mouse Nkx2-3 homeodomain gene is expressed in gut mesenchyme during pre- and postnatal mouse development.. Dev Dyn.

[21] Reecy JM, Yamada M, Cummings K, Sosic D, Chen CY (1997). Chicken Nkx-2.8: a novel homeobox gene expressed in early heart progenitor cells and pharyngeal pouch-2 and -3 endoderm.. Dev Biol.

[22] Brand T, Andrée B, Schneider A, Buchberger A, Arnold HH (1997). Chicken Nkx2-8, a novel homeobox gene expressed during early heart and foregut development.. Mech Dev.

[23] Newman CS, Krieg PA (1998). tinman-related genes expressed during heart development in Xenopus.. Dev Genet.

[24] Fu Y, Yan W, Mohun TJ, Evans SM (1998). Vertebrate tinman homologues XNkx2-3 and XNkx2-5 are required for heart formation in a functionally redundant manner.. Development.

[25] Garrity DM, Childs S, Fishman MC (2002). The heartstrings mutation in zebrafish causes heart/fin Tbx5 deficiency syndrome.. Development.

[26] Targoff KL, Schell T, Yelon D (2008). Nkx genes regulate heart tube extension and exert differential effects on ventricular and atrial cell number.. Dev Biol.

[27] Walsh EC, Stainier DY (2001). UDP-glucose dehydrogenase required for cardiac valve formation in zebrafish.. Science.

[28] Hurlstone AF, Haramis AP, Wienholds E, Begthel H, Korving J (2003). The Wnt/beta-catenin pathway regulates cardiac valve formation.. Nature.

[29] Huang H, Zhang B, Hartenstein PA, Chen JN, Lin S (2005). NXT2 is required for embryonic heart development in zebrafish.. BMC Dev Biol.

[30] Prall OW, Menon MK, Solloway MJ, Watanabe Y, Zaffran S (2007). An Nkx2-5/Bmp2/Smad1 negative feedback loop controls heart progenitor specification and proliferation.. Cell.

[31] Vaughan CJ, Basson CT (2000). Molecular determinants of atrial and ventricular septal defects and patent ductus arteriosus.. Am J Med Genet.

[32] Liberatore CM, Searcy-Schrick RD, Yutzey KE (2000). Ventricular expression of tbx5 inhibits normal heart chamber development.. Dev Biol.

[33] Westerfield M (1995). The zebrafish book.

[34] Huang CJ, Tu CT, Hsiao CD, Hsieh FJ, Tsai HJ (2003). Germ-line transmission of a myocardium-specific GFP transgene reveals critical regulatory elements in the cardiac myosin light chain 2 promoter of zebrafish.. Dev Dyn.

[35] Kimmel CB, Ballard WW, Kimmel SR, Ullmann B, Schilling TF (1995). Stages of embryonic development of the zebrafish.. Dev Dyn.

[36] Chen YH, Lee WC, Liu CF, Tsai HJ (2001). Molecular structure, dynamic expression, and promoter analysis of zebrafish (Danio rerio) myf-5 gene.. Genesis.

[37] Martínez-Barberá JP, Toresson H, Da Rocha S, Krauss S (1997). Cloning and expression of three members of the zebrafish Bmp family: Bmp2a, Bmp2b and Bmp4.. Gene.

[38] Szeto DP, Griffin KJ, Kimelman D (2002). HrT is required for cardiovascular development in zebrafish.. Development.

[39] Yelon D, Horne SA, Stainier DY (1999). Restricted expression of cardiac myosin genes reveals regulated aspects of heart tube assembly in zebrafish.. Dev Biol.

[40] Lin CY, Yung RF, Lee HC, Chen WT, Chen YH (2006). Myogenic regulatory factors Myf5 and Myod function distinctly during craniofacial myogenesis of zebrafish.. Dev Biol.

[41] Moore JL, Aros M, Steudel KG, Cheng KC (2002). Fixation and decalcification of adult zebrafish for histological, immunocytochemical, and genotypic analysis.. Biotechniques.

